# Supplementary material for: Actin dysregulation induces neuroendocrine plasticity and immune evasion: a vulnerability of small cell lung cancer
Source: Nat Commun. 2025 Dec 6;17:386. doi: 10.1038/s41467-025-67078-9 (PMC12795832; doi:10.1038/s41467-025-67078-9)
Supplement: Supplementary file 1 — Supplementary Information File [file 41467_2025_67078_MOESM1_ESM.pdf]

List of Supplementary Materials

Supplementary Figures 1 to 12

Supplementary Figures

Supplementary Figure 1. CRACD inactivation in SCLC
Supplementary Figure 2. scRNA-seq of preSC (*Cracd* WT vs. KO) allograft tumors
Supplementary Figure 3. scRNA-seq of RPR2 vs. CRPR2 SCLC tumors
Supplementary Figure 4. scRNA-seq analysis of pathway-associated gene expression
Supplementary Figure 5. NOTCH signaling downregulation by *Cracd* KO
Supplementary Figure 6. Immune cell profiling of RPR2 vs. CRPR2 SCLC tumors
Supplementary Figure 7. scRNA-seq-based immune cell profiling
Supplementary Figure 8. Impact of EZH2 blockade on Cracd-inactivated SCLC tumorigenesis
Supplementary Figure 9. Gene expression and pathway enrichment analysis
Supplementary Figure 10. scRNA-seq analysis of the human SCLC tumor datasets
Supplementary Figure 11. Immunohistochemical staining in human SCLC tissues
Supplementary Figure 12. Uncropped immunoblot images

Supplementary Reference

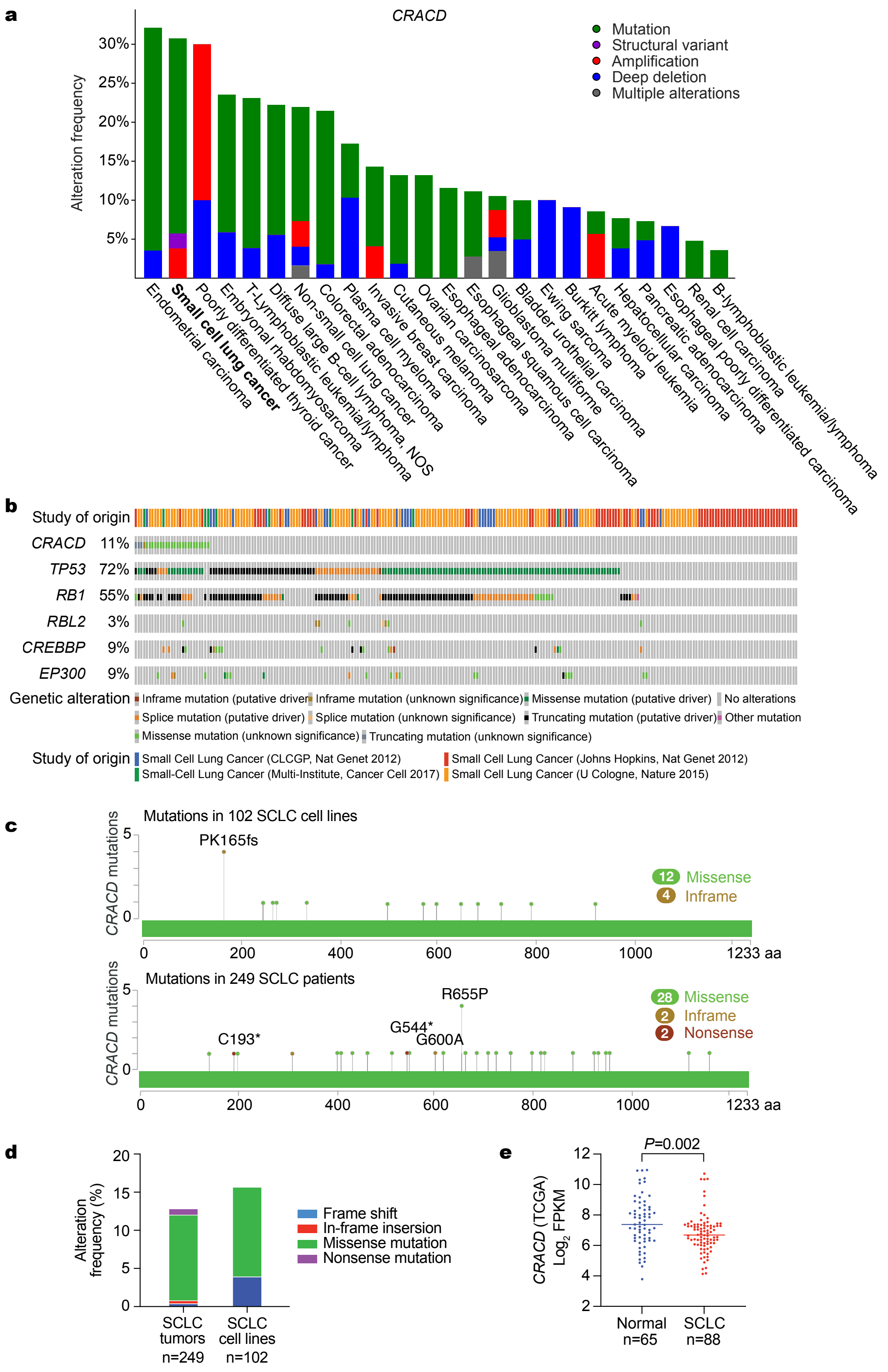

Supplementary Figure 1. CRACD inactivation in SCLC

a. Somatic alterations in *CRACD*, *TP53*, *RB1*, *RBL2*, *CREBBP*, and *EP300* in 249 SCLC patient samples. The diagram was generated using OncoPrinter (www.cbioportal.org/oncoprinter.jsp).

b. Types of *CRACD* mutations found in SCLC patients and cell lines.

c, d. Distribution and frequency of *CRACD* mutations found in 102 SCLC cell lines and 249 SCLC patient samples, illustrated along the length of the protein and summarized by mutation type (frame shift, in-frame insertion, missense, and nonsense). The diagram was generated using the Broad Institute Cancer Cell Line Encyclopedia [CCLE] and cBioPortal databases.

e. Levels of *CRACD* mRNA transcripts in normal lung and SCLC patient tumor samples from the TCGA database; Student's *t*-test.

Source data are provided as a Source Data file.

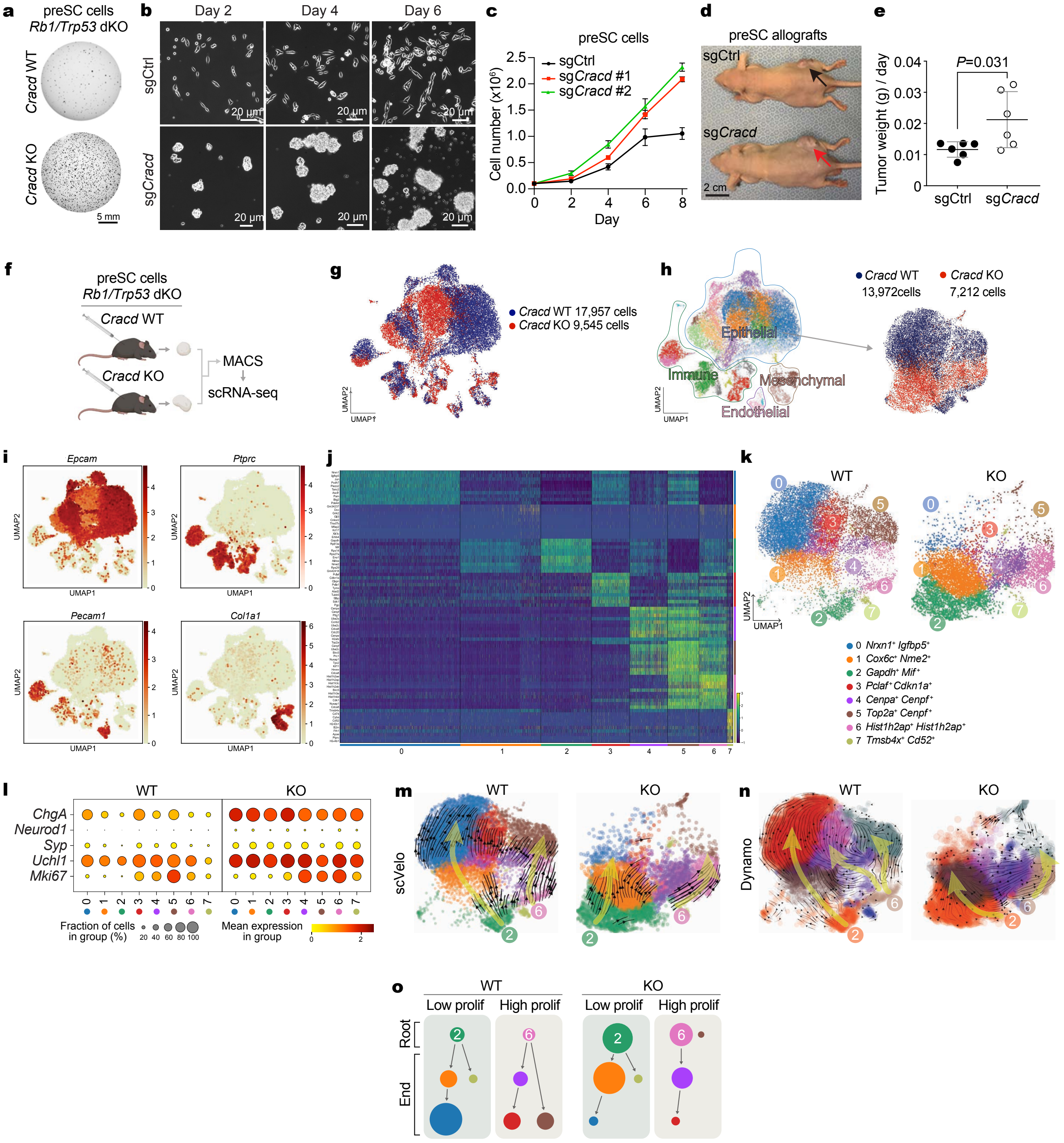

Supplementary Figure 2. scRNA-seq of preSC (*Cracd* WT vs. KO) allograft tumors

a-c. Mouse preSCs were engineered to target Cracd alleles using CRISPR-mediated gene editing and characterized for cell morphology (a), short-term proliferation (b), and colony-forming ability (c). Scale bars: 20 µm.

d. Nude mice 40 days after injection of preSCs (*Cracd* WT vs. KO). Images of allograft tumors (arrows) derived from preSCs in the flanks of athymic nude mice.

e. Quantification of tumor development (tumor weight/days taken to reach endpoint) in the allograft model.
f. Experimental scheme of the workflow for preSC allograft transplantation, tumor dissociation, single cell isolation, and scRNA-seq; magnetic-activated cell sorting (MACS).
g. UMAP of integrated scRNA-seq datasets of allograft tumor generated from *Cracd* WT vs. KO preSCs.
h. UMAPs of subsets of cells from the global level (left) to the allograft tumor cells (right). Each dot represents a single cell, colored by cell type.
i. Feature plots of mouse cell-type marker gene expression: *Epcam* (epithelial cells), *Ptprc* (immune cells), *Pecam1* (endothelial cells), and *Colla1* (mesenchymal cells).
j. Heatmap of gene expression-based cell clusters. The top 10 genes that were highly expressed in each cell cluster were visualized; these were used for cell annotation of *Cracd* WT and KO allograft tumors.
k. Uniform Manifold Approximation and Projection (UMAP) plots of cell types within *Cracd* WT (left) and preSC *Cracd* KO allograft tumors (right).
l. Dot plot depicting selected gene expression between each cell cluster in *Cracd* WT and *Cracd* KO preSC allograft tumors. Dot size, percentage of cells expressing gene; dot color, mean expression scaled from 0-2.5.
m, n. Cell lineage trajectory inference analysis by using scVelo (i) and Dynamo (j)
o. Illustration of cell lineages of preSC tumors. Representative images (n >= 3) are shown; P values were calculated using Student's *t*-test; error bars: standard deviation (SD). Panel f was created with BioRender.com.
Source data are provided as a Source Data file.

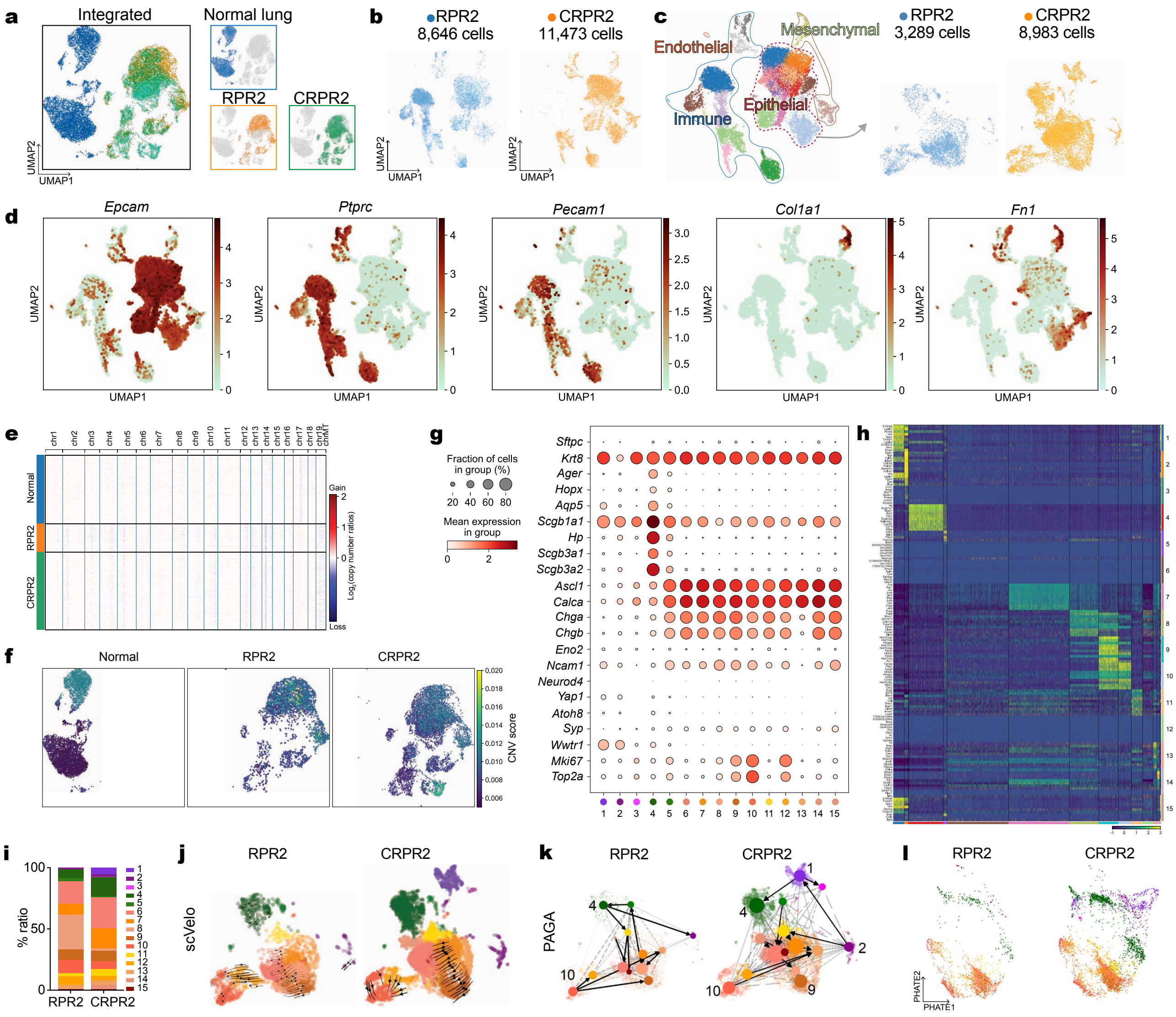

Supplementary Figure 3. scRNA-seq of RPR2 vs. CRPR2 SCLC tumors

a-b. UMAPs of the scRNA-seq datasets of normal mouse lung, RPR2, and CRPR2 SCLC tumors.

c. Subset UMAPs showing global cell populations and tumor epithelial subsets. Each dot represents a single cell colored by cell type.

d. Feature plots displaying the expression of each representative marker: *Epcam* (epithelial cells), *Ptprc* (immune cells), *Pecam1* (endothelial cells), *Colla1* (mesenchymal cells), and *Fn1* (mesenchymal cells).

e, f. Copy number variation (CNV) analysis showing genomic gains/losses in RPR2 and CRPR2 tumors compared with normal lung (e). CNV scores projected onto UMAPs (f).

g. Dot plots for mouse lung epithelial marker gene expression in each cell cluster.

h. Heatmap of each cell cluster of RPR2 and CRPR2 tumors. The top 10 genes that were highly expressed in each cell cluster were visualized; these were used for cell annotation.

i. Comparison of proportions of different cell types between the RPR2 and CRPR2 datasets.

j. RNA velocity–based cell lineage trajectory analysis of RPR2 and CRPR2 scRNA-seq datasets. RNA velocity was calculated through a dynamic model, and cells were clustered using the “Leiden” algorithm, the scVelo and Scanpy packages (n_neighbors = 10, n_pcs = 40).

k. Partition-based graph abstraction–based visualization of cell lineage trajectories of RPR2 and CRPR2 tumor cells. The size of the circle corresponds to the cell number. A partition-based graph abstraction analysis was performed and plotted using the RNA velocity–based cell clusters (scVelo). While clusters 4 and 10 in RPR2 tumors were inferred as root cell clusters, CRPR2 tumors likely harbored three additional root cell clusters (1, 2, and 9), repopulating tumor cells (also see Supplementary Video S1). Among those, cluster 1 was exclusively found in *Cracd* KO tumors (CRPR2). It should be noted that cell lineage trajectories were inferred by RNA splicing (RNA velocity) of scRNA-seq datasets.

l. PHATE mapping of RPR2 and CRPR2 scRNA-seq datasets.

Source data are provided as a Source Data file.

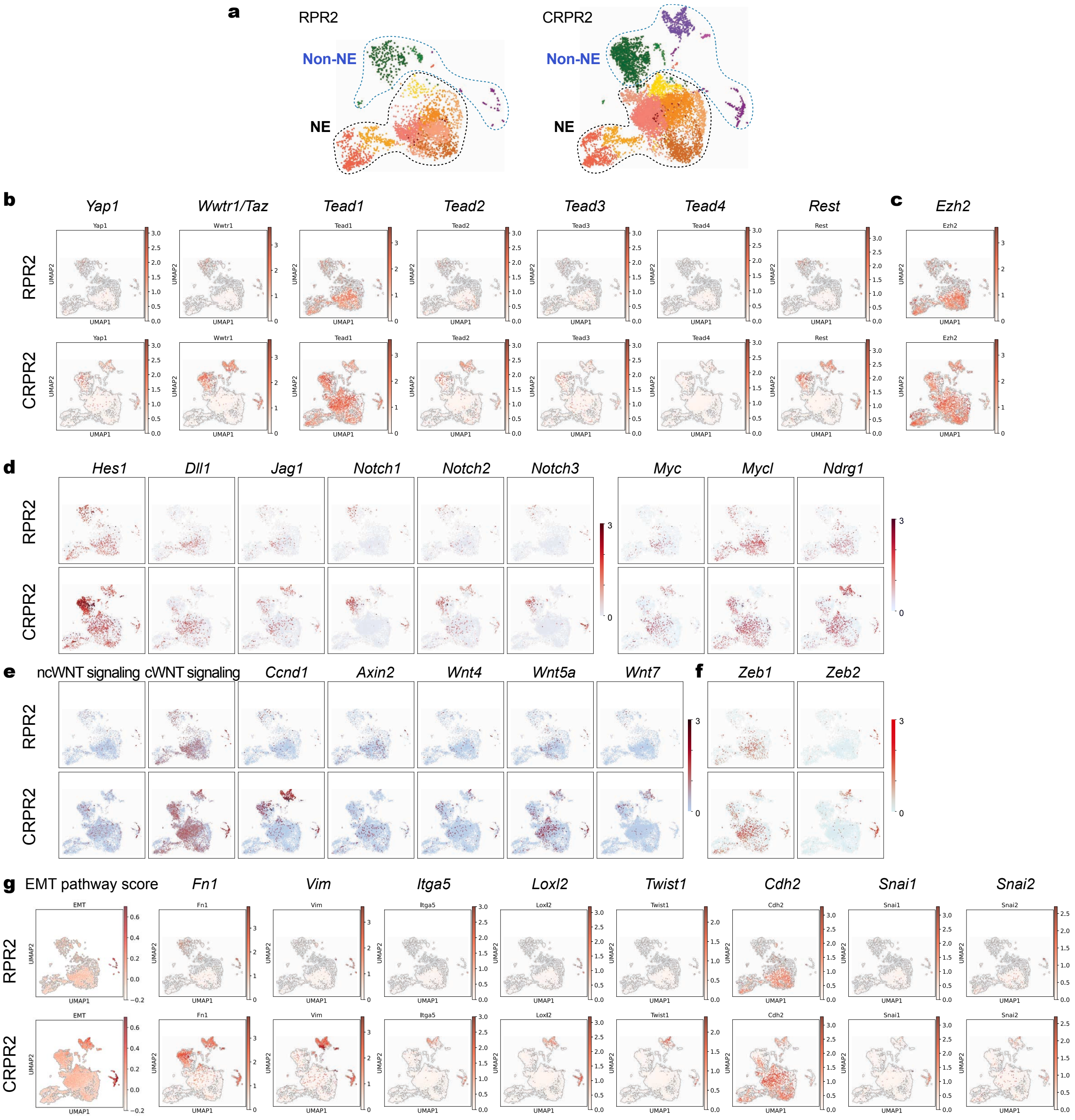

Supplementary Figure 4. scRNA-seq analysis of pathway-associated gene expression

a. UMAP plots of tumor cells from RPR2 and CRPR2 tumors, colored by cluster identity. NE and non-NE cells are annotated by dashed outlines.

b-g. Feature plots showing normalized expression of genes associated with the indicated pathways in RPR2 and CRPR2 tumors. (b) Hippo pathway-associated genes: *Yap1, Wwtr1/Taz, Tead1, Tead2, Tead3, Tead4*, and *Rest*. (c) *Ezh2*. (d) NOTCH signaling-associated genes: *Hes1, Dll1, Jag1, Notch1, Notch2, Notch3, Myc, Mycl, and Ndrg1*. (e) WNT signaling-associated genes (*Ccnd1, Axin2, Wnt4,*

Wnt5a, Wnt7) and WNT pathway score. (f) EMT transcription factors: *Zeb1 and Zeb2*. (g) EMT pathway score and EMT-related genes: *Fn1, Vim, Itga5, Loxl2, Twist1, Cdh2, Snai1, and Snai2*.

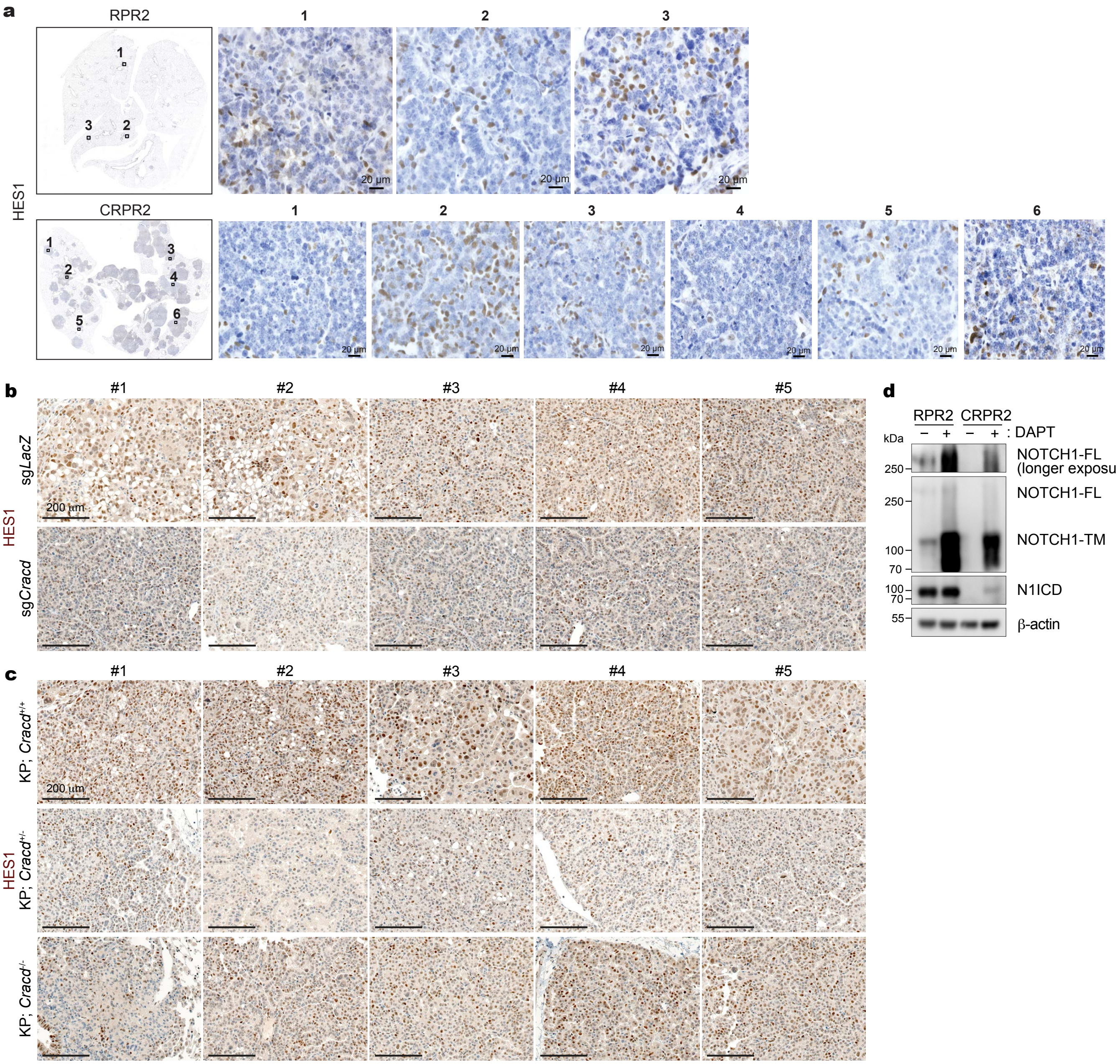

Supplementary Figure 5. NOTCH signaling downregulation by *Cracd* KO

a. IHC of murine lungs (RPR2 vs. CRPR2) for HES1.

b. IHC of murine lungs isolated from LUAD models (*Kras*^{LSLG12D}, *Trp53*$^{floxed/floxed}$ [KP]) instilled with adenovirus encoding Cre recombinase, Cas9, and sgRNAs (*LacZ* [control] or *Cracd*), showing the impact of conditional KO of *Cracd* on KP-driven LUAD tumorigenesis, as we recently performed[1].

c. IHC of murine lungs isolated from LUAD models (*Kras*^{LSLG12D}, *Trp53*$^{floxed/floxed}$ [KP] or *Cracd*$^{-/-}$, *Kras*^{LSLG12D}, *Trp53*$^{floxed/floxed}$ [CKP]) instilled with adenovirus encoding Cre recombinase, displaying the impact of germline KO of *Cracd* on KP-driven LUAD tumorigenesis, as we recently performed[1].

d. IB of RPR2 or CRPR2 cells treated with DAPT, a gamma-secretase inhibitor (10 uM, 48hrs). FL: full length, TM: transmembrane.

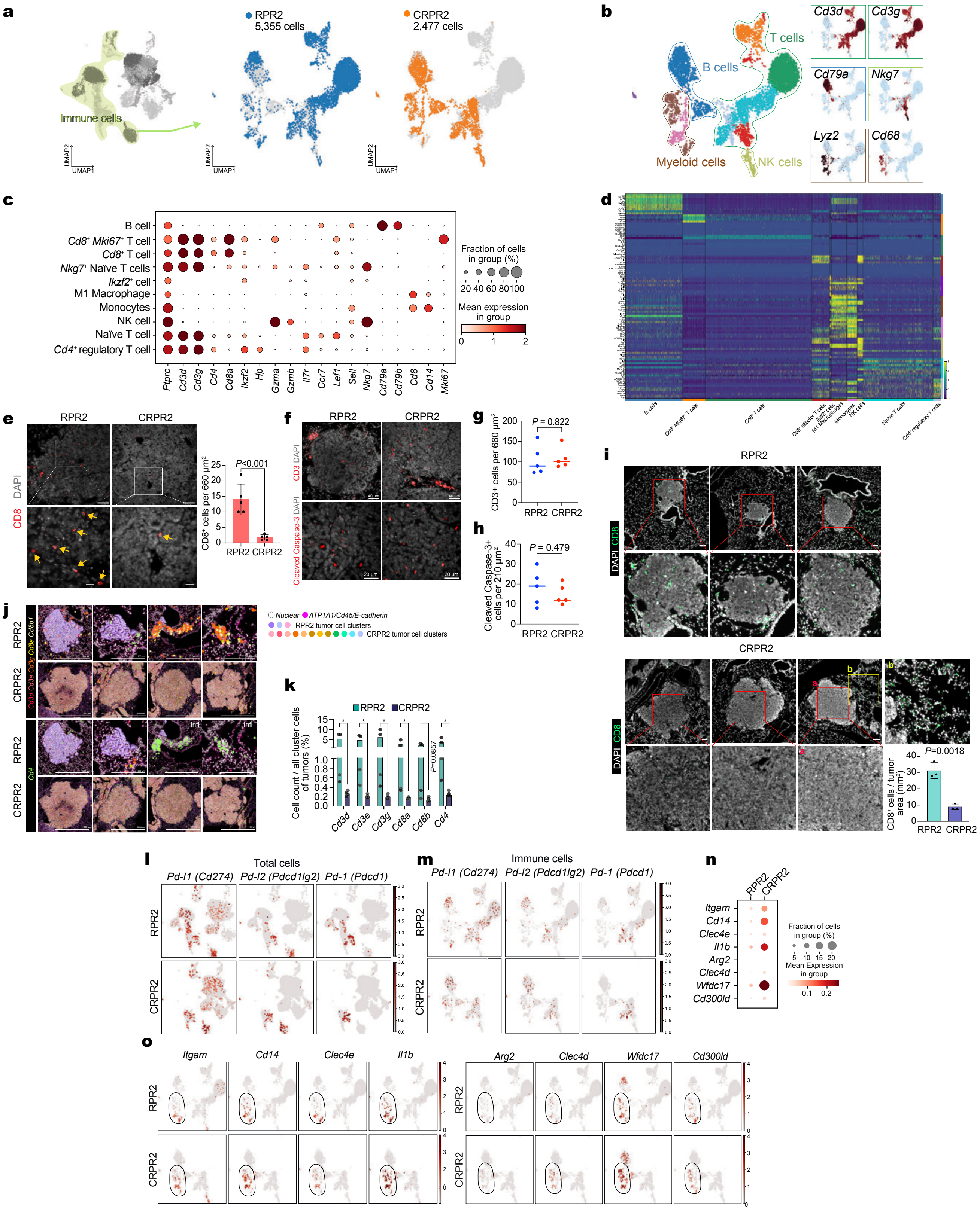

Supplementary Figure 6. Immune cell profiling of RPR2 vs. CRPR2 SCLC tumors

a. UMAPs showing global and immune-cell subsets from RPR2 and CRPR2 scRNA-seq datasets. Each dot represents one cell colored by cell type.

b. Re-clustered immune cells with marker expression: *Cd3d*/*Cd3g* (T cells), *Cd79a* (B cells), *Nkg7* (NK cells), Lyz2/Cd68 (myeloid).

c, d. Dot plot and heatmap of top immune-cell marker genes across clusters in integrated *Cracd* WT and KO datasets.

e, h. Immunostaining for CD8 and CD3 in RPR2 and CRPR2 tumors showing reduced intratumoral $CD8^+$ T cells but no change in total T-cell number or apoptosis. Scale bars, 50 μm. Representative images are shown. *P* values were calculated using the Student's *t*-test; error bars: SD.

i. Analysis of peritumoral $CD8^+$ T cells. Immunostaining of the tumor margins in RPR2 and CRPR2 tumors. The top panels show low-magnification views with red boxes indicating regions of interest. Bottom panels show higher-magnification images of the indicated ROIs. Panels labeled (a) and (b) show enlarged views of selected areas, highlighting CD8+ T cell distribution. Quantification of CD8+ T cell density per unit area in tumor sections. Data represent mean ± SEM from n=3 tumors per group. *P* values were calculated using the Student's *t*-test; error bars: SD.

j, k. Spatial localization of T cells (*Cd3d, Cd3e, Cd3g, Cd8a, Cd8b1, Cd4*) identified by Xenium transcriptomic signatures (n), quantification of T cells density per unit area from Xeinum (o). Statistical significance was assessed by Student's *t*-test.

l, m. Feature plots of *Pd-l1*, *Pd-l2*, and *Pd-1* expression in all cells between RPR2 and CRPR2 scRNA-seq datasets (j). Feature plots of *Pd-l1*, *Pd-l2*, and *Pd-1* expression in immune cells between RPR2 and CRPR2 datasets (k).

n. Dot plot showing the expression level of MDSC marker genes (*Itgam*, *Cd14*, *Clec4d/e*, *Il1b*, *Arg2*, *Wfdc17*, and *Cd300ld*) in RPR2 and CRPR2 datasets.

o. Feature plots of MDSC marker gene expression in immune cells between the RPR2 and CRPR2 datasets. *Cracd* KO upregulates the expression of MDSC markers (*Itgam*, *Cd14*, *Clec4d/e*, *Il1b*, *Arg2*, *Wfdc17*, and *Cd300ld*). Circle, myeloid cell cluster.

Source data are provided as a Source Data file.

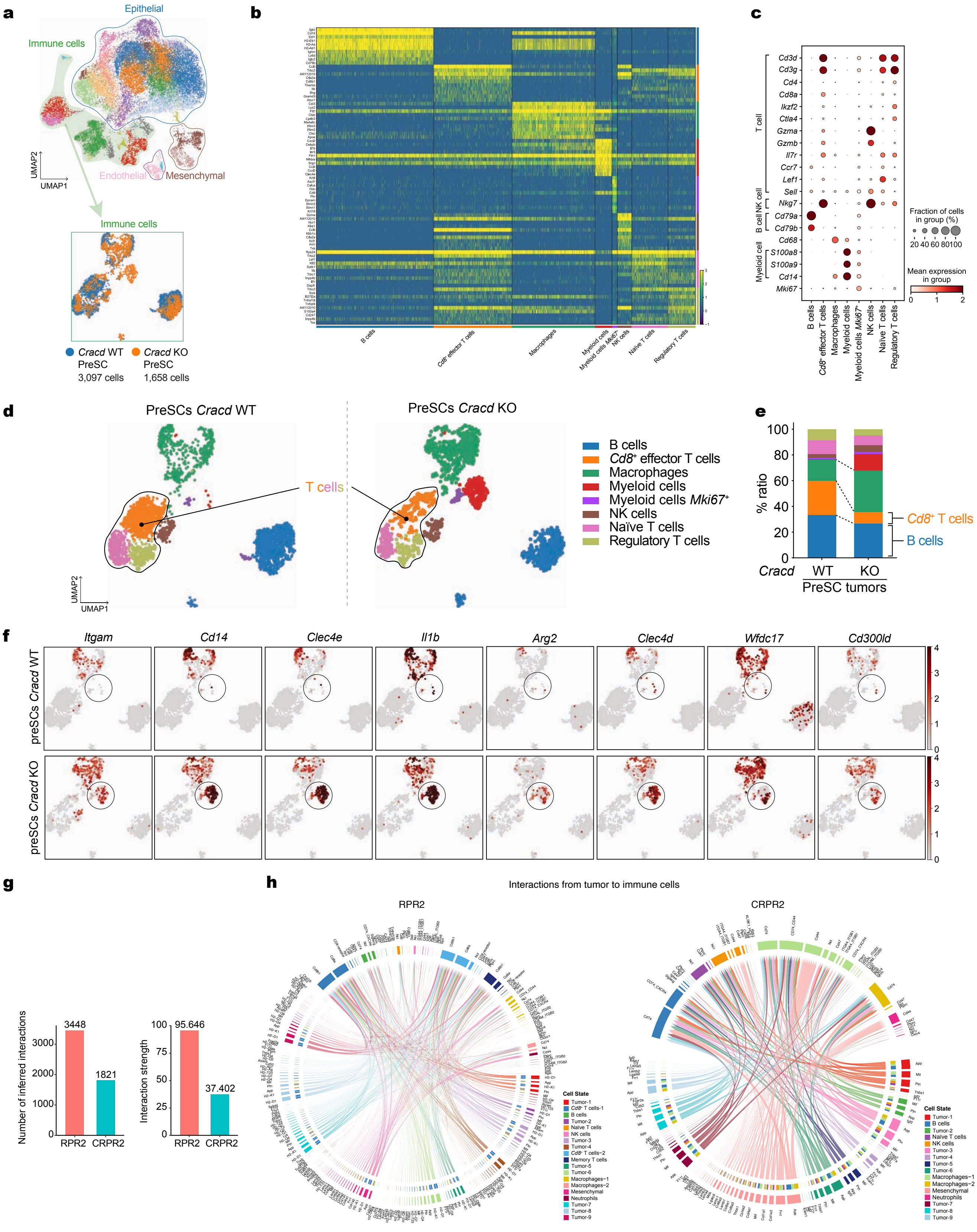

Supplementary Figure 7. scRNA-seq-based immune cell profiling

a. UMAP of subsets of cells from the global level (upper) and immune cell subsets (lower) in integrated scRNA-seq datasets of preSC allograft tumors (*Cracd* WT vs. KO). Each dot represents a single cell, colored by cell type.

b. Heatmap of gene expression–based cell clusters of the top 10 genes that were most highly expressed in each immune cell type of integrated scRNA-seq datasets of preSC allograft tumors (*Cracd* WT vs. *Cracd* KO).

c. Dot plots show mouse immune cell marker gene expression in each cell cluster.

d. *Cracd* KO decreases the number of $Cd8^+$ effector T cells in preSC tumors. UMAPs of preSC *Cracd* WT (left) and preSC *Cracd* KO (right) subsets showing each immune cell type.

e. Cell proportion analysis of different immune cell types between preSC *Cracd* WT and KO datasets.

f. Dot plot showing the upregulation of MDSC markers expression by *Cracd* KO preSC tumors compared to *Cracd* WT preSC tumors. Circle, myeloid cell cluster.

g. Total cell-cell interactions (left) and interaction strength (right) from RPR2 and CRPR2 tumors were analyzed using the CellChat package.

h. Cell-cell interaction analysis with CellChat. Chord plots show significant changes in signaling between immune cells in the RPR2 (left) and CRPR2 (right) SCLC tumors. The inner bar colors represent the cell clusters that receive signals. The inner bar size is proportional to the signal strength received by the cell clusters. Chords indicate ligand-receptor pairs that mediate the interaction between two cell clusters; the size of the chords is proportional to the signal strength of the given ligand-receptor pair. RPR2 tumors showed a strong interaction between the MHC-I pathway (*H2-K*, *H2-D*, *H2-Q*, and *H2-T* from all tumor cell clusters) and CD8 receptors (from $CD8^+$ T cells). In contrast, CRPR2 tumors exhibited no interaction between the MHC-I pathway and CD8 receptors. Intriguingly, in CRPR2 tumors, significant cell-cell interactions include tumor cells (via App, Mif, and Ptn)-B cells (*Cd74_Cxcr4*), Naïve T cells (*Ncl*, *Cd47*), NK cells (*Ncl*, *Itga4*, *Cd44*, and *Cd47*), and macrophages (*Cd74*, *Cd44*, *Ncl*, and *Itga4*).

Source data are provided as a Source Data file.

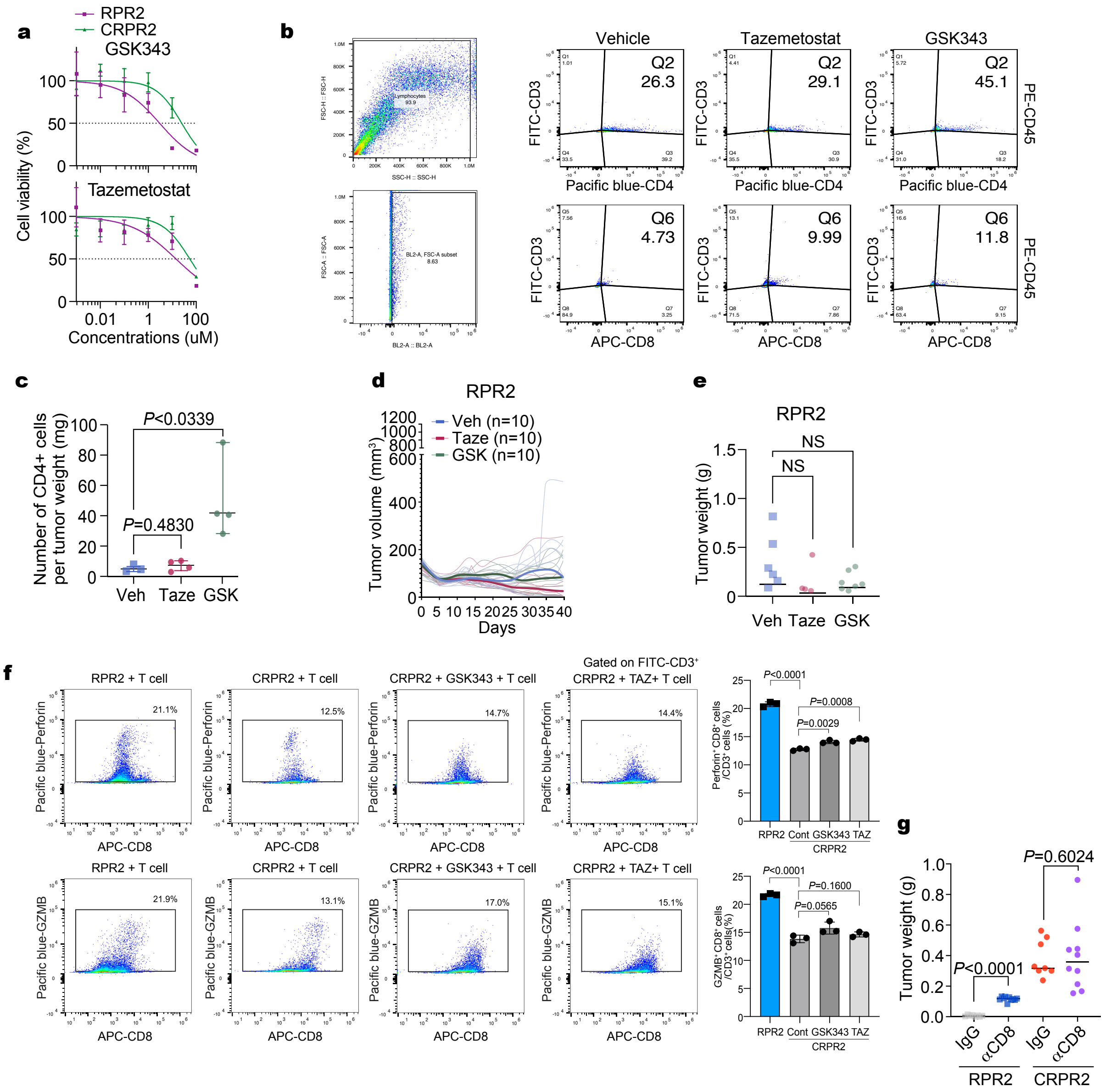

Supplementary Figure 8. Impact of EZH2 blockade on SCLC tumorigenesis

- **a.** Cell viability assay of RPR2 and CRPR2 cell lines treated with EZH2 inhibitors, GSK343 and Tazemetostat. Cell viability was measured by Cell Counting Kit-8.
- **b.** Flow cytometry analysis using FlowJo to assess T cell populations in CRPR2 tumors treated with vehicle (Veh), Tazemetostat (Taze), or GSK343 (GSK). Representative plots show pacific blue-CD4 or APC-CD8 T cell populations within PE-CD45 and FITC-CD3 double-positive cells. Q2 and Q6 quadrants indicate the percentage of positive cells.
- **c.** Quantification of $CD4^+$ T cells per tumor weight in CRPR2 tumors treated with EZH2 inhibitors. The total number of $CD4^+$ T cells was quantified from freshly isolated tumors and normalized by tumor weight (mg) to account for tumor size differences.
- **d.** Tumor growth curves of RPR2 subcutaneous tumors in immunocompetent mice treated with vehicle (Veh), Tazemetostat (Taze; 200 mg/kg, oral gavage), or GSK343 (GSK; 20mg/kg, intraperitoneal), administered every other day (n=10 per group). Tumor volume was measured every other day.
- **e.** Tumor growth was subsequently assessed by measuring tumor weight. No statistical significance was observed; NS, not significant by Student's *t*-test ($P \geq 0.05$).
- **f.** CD8+ T cells were co-cultured with CRPR2 or RPR2 cells, and intracellular expression of Perforin and Granzyme B was assessed by flow cytometry. *P*-values are indicated.

g. Tumors were harvested and weighted at day 21 post subcutaneous injection. Each dot represents an individual tumor. C57BL/6 mice were subcutaneously injected with RPR2 or CRPR2 cells and treated with anti-CD8 antibody. Statistical significance was determined by Student's *t*-test.

Source data are provided as a Source Data file.

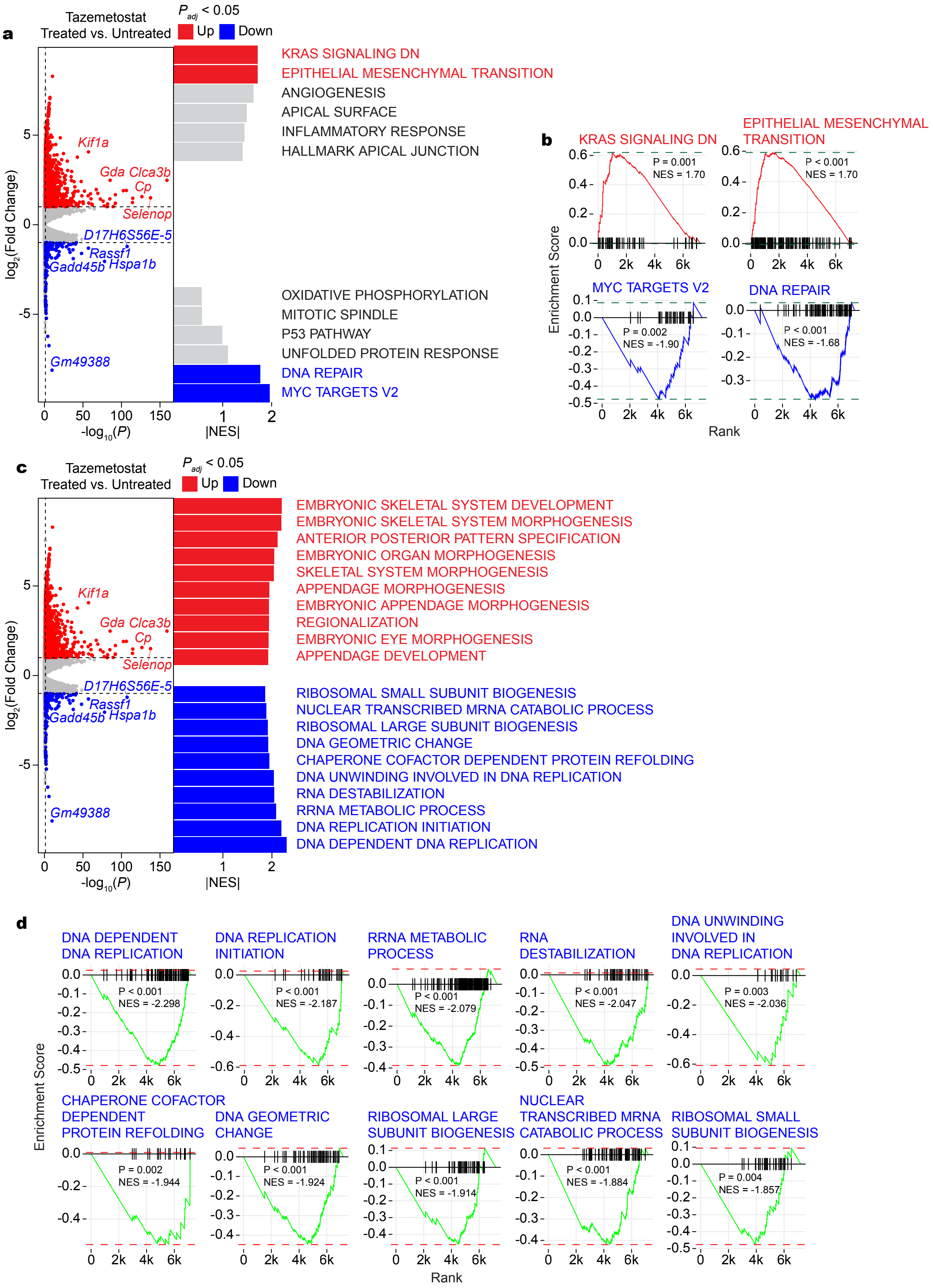
a
Tazemetostat Treated vs. Untreated
P_{adj} < 0.05
Up
Down
KRAS SIGNALING DN
EPITHELIAL MESENCHYMAL TRANSITION
ANGIOGENESIS
APICAL SURFACE
INFLAMMATORY RESPONSE
HALLMARK APICAL JUNCTION
OXIDATIVE PHOSPHORYLATION
MITOTIC SPINDLE
P53 PATHWAY
UNFOLDED PROTEIN RESPONSE
DNA REPAIR
MYC TARGETS V2
Kif1a
Gda Clca3b
Cp
Selenop
D17H6S56E-5
Rassf1
Gadd45b Hspa1b
Gm49388
log2(Fold Change)
-log10(P)
|NES|
b
KRAS SIGNALING DN
P = 0.001
NES = 1.70
EPITHELIAL MESENCHYMAL TRANSITION
P < 0.001
NES = 1.70
MYC TARGETS V2
P = 0.002
NES = -1.90
DNA REPAIR
P < 0.001
NES = -1.68
Enrichment Score
Rank
c
Tazemetostat Treated vs. Untreated
P_{adj} < 0.05
Up
Down
EMBRYONIC SKELETAL SYSTEM DEVELOPMENT
EMBRYONIC SKELETAL SYSTEM MORPHOGENESIS
ANTERIOR POSTERIOR PATTERN SPECIFICATION
EMBRYONIC ORGAN MORPHOGENESIS
SKELETAL SYSTEM MORPHOGENESIS
APPENDAGE MORPHOGENESIS
EMBRYONIC APPENDAGE MORPHOGENESIS
REGIONALIZATION
EMBRYONIC EYE MORPHOGENESIS
APPENDAGE DEVELOPMENT
RIBOSOMAL SMALL SUBUNIT BIOGENESIS
NUCLEAR TRANSCRIBED MRNA CATABOLIC PROCESS
RIBOSOMAL LARGE SUBUNIT BIOGENESIS
DNA GEOMETRIC CHANGE
CHAPERONE COFACTOR DEPENDENT PROTEIN REFOLDING
DNA UNWINDING INVOLVED IN DNA REPLICATION
RNA DESTABILIZATION
RRNA METABOLIC PROCESS
DNA REPLICATION INITIATION
DNA DEPENDENT DNA REPLICATION
log2(Fold Change)
-log10(P)
|NES|
d
DNA DEPENDENT DNA REPLICATION
P < 0.001
NES = -2.298
DNA REPLICATION INITIATION
P < 0.001
NES = -2.187
RRNA METABOLIC PROCESS
P < 0.001
NES = -2.079
RNA DESTABILIZATION
P < 0.001
NES = -2.047
DNA UNWINDING INVOLVED IN DNA REPLICATION
P = 0.003
NES = -2.036
CHAPERONE COFACTOR DEPENDENT PROTEIN REFOLDING
P = 0.002
NES = -1.944
DNA GEOMETRIC CHANGE
P < 0.001
NES = -1.924
RIBOSOMAL LARGE SUBUNIT BIOGENESIS
P < 0.001
NES = -1.914
NUCLEAR TRANSCRIBED MRNA CATABOLIC PROCESS
P < 0.001
NES = -1.884
RIBOSOMAL SMALL SUBUNIT BIOGENESIS
P = 0.004
NES = -1.857
Enrichment Score
Rank

Supplementary Figure 9. Gene expression and pathway enrichment analysis

a. Volcano plot showing DEGs between Tazemetostat-treated and untreated CRPR2 organoids. Genes with adjusted $P < 0.05$ are highlighted (red: upregulated, blue: downregulated). Enriched HALLMARK gene sets among up- or downregulated genes are shown as bar plots on the right.

b. GSEA plots for selected HALLMARK gene sets shown in (a). NES and *P*-values are indicated.

c. Volcano plot showing DEGs between Tazemetostat-treated and untreated CRPR2 organoids. Genes with adjusted $P < 0.05$ are highlighted (red: upregulated, blue: downregulated). Enriched Gene Ontology (Biological Process) GOBP gene sets among up- or downregulated genes are shown as bar plots on the right.

d. GSEA plots for selected GOBP gene sets shown in (a). NES and *P*-values are indicated.

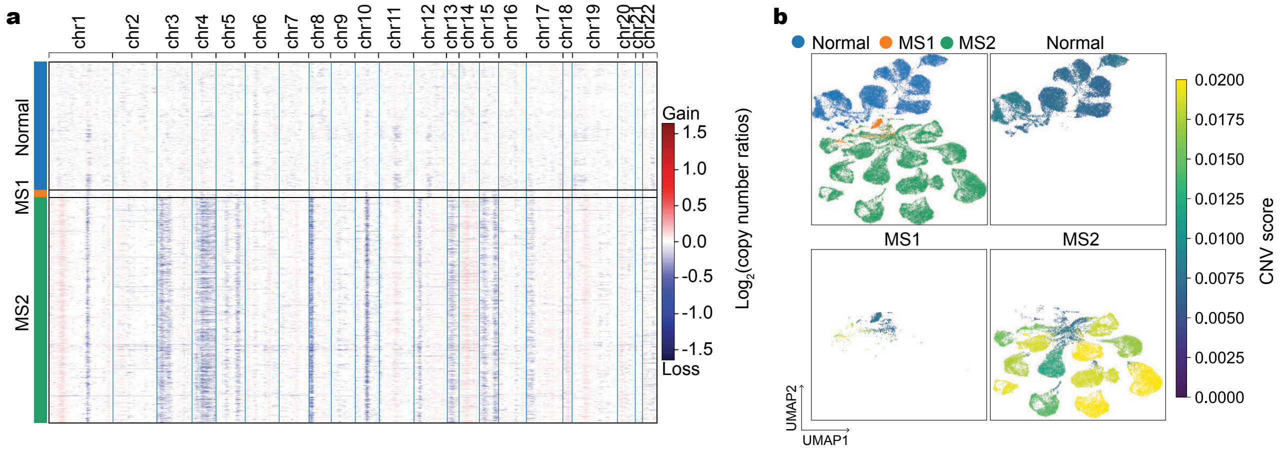

Supplementary Figure 10. scRNA-seq analysis of the human SCLC tumor datasets

a, b. A copy number variation analysis of MS1 and MS2 tumors. Copy number variation plot showing the distribution of genomic alterations (gains and losses) in MS1 and MS2 tumors compared with healthy human lung samples (a). Copy number variation scores were projected into the UMAP of the scRNA-seq dataset from healthy human lung samples and MS1/2 samples (b).

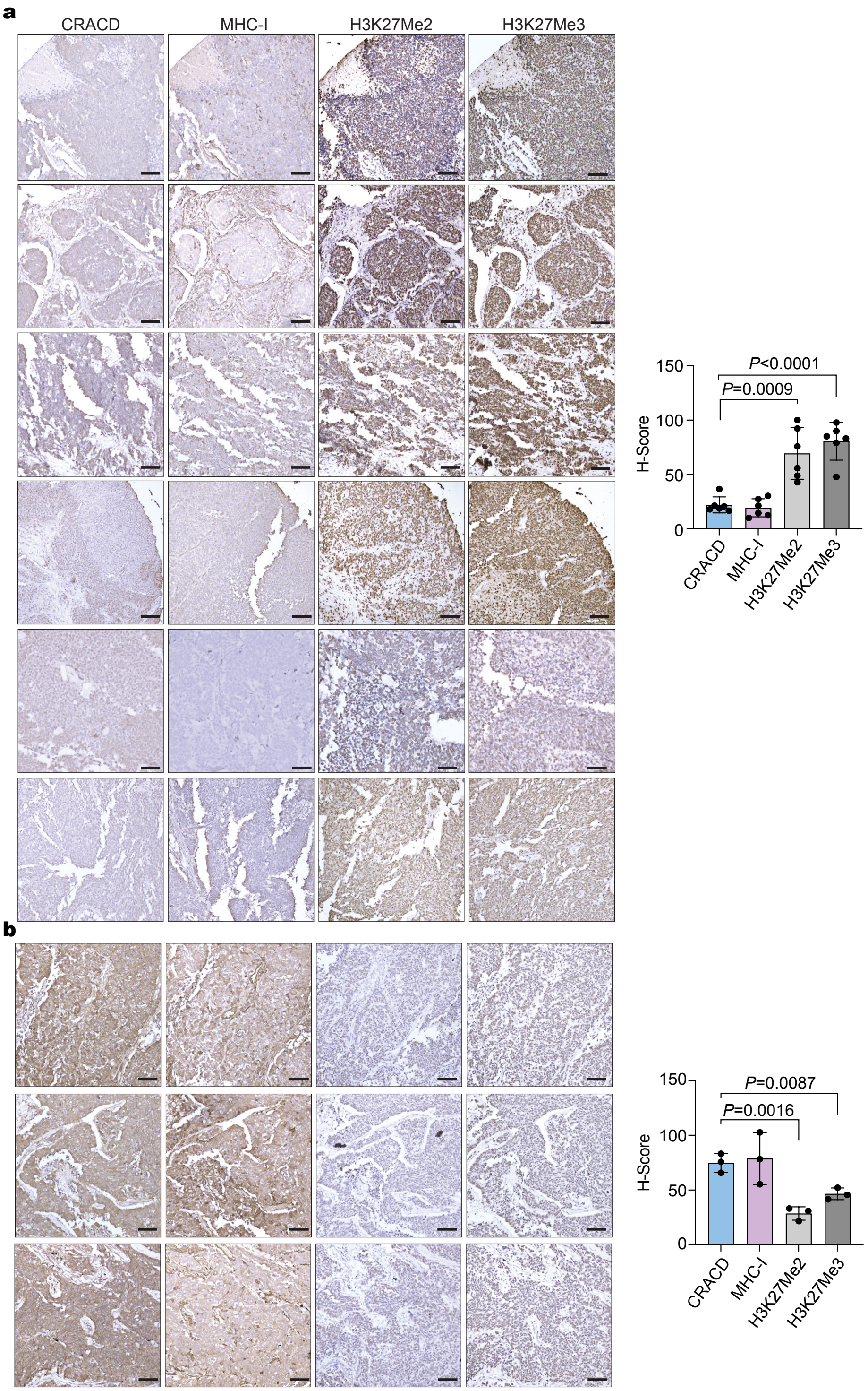

Supplementary Figure 11. Immunohistochemical staining in human SCLC tissues

a, b. Immunohistochemical staining of CRACD, MHC-I, H3K27Me2, and H3K27Me3 in human SCLC tissues. Tumor sections were grouped based on CRACD expression status, and corresponding changes in MHC-I and histone methylation marks were examined. Quantification was performed using H-score analysis. Statistical significance was assessed by Student's *t*-test. Data are presented as mean ± SEM; $P < 0.05$ was considered significant.

Source data are provided as a Source Data file.

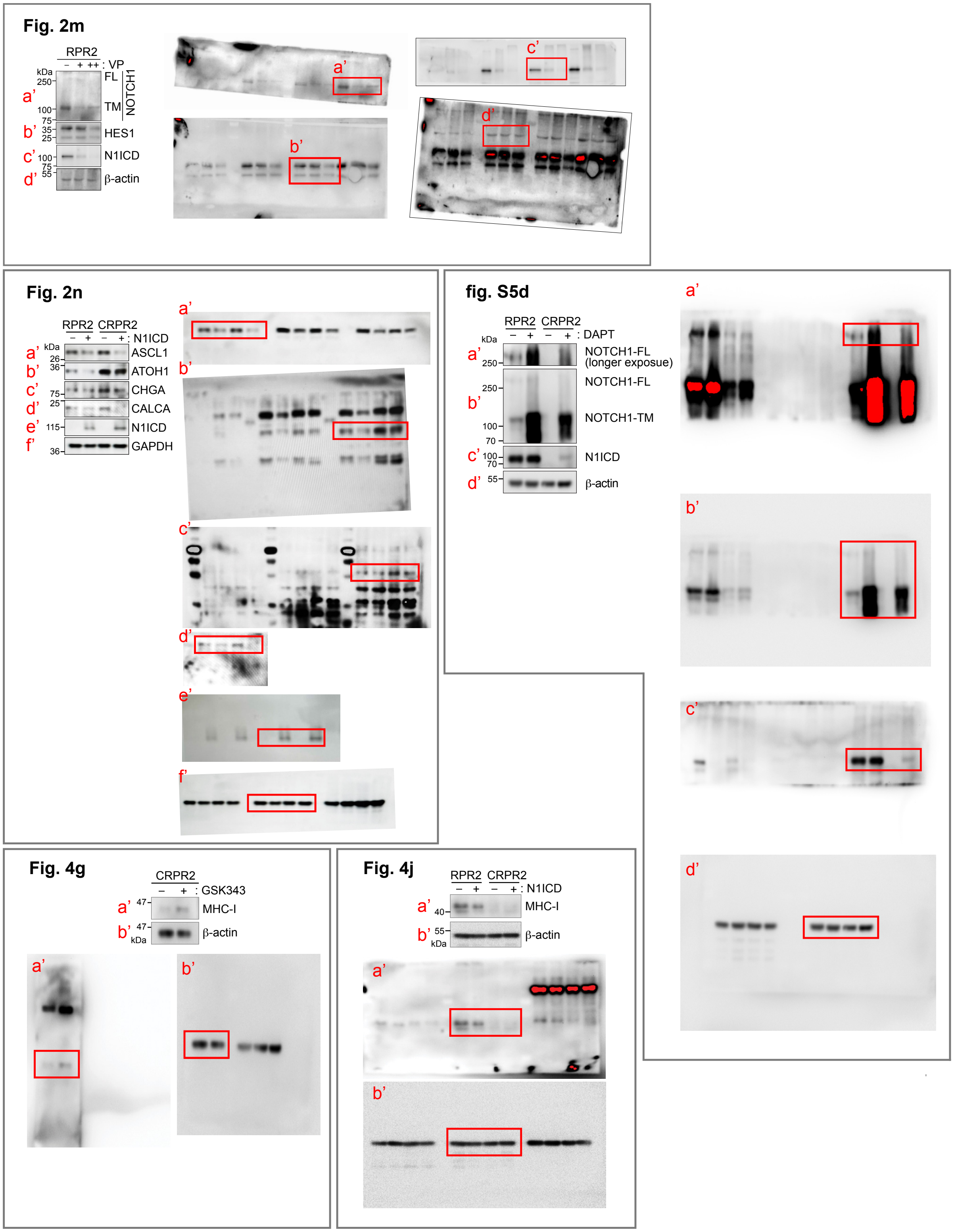

Supplementary Figure 12. Uncropped immunoblot images

Supplementary reference

1. Kim B, Zhang S, Huang Y, Ko KP, Jung YS, Jang J, Zou G, Zhang J, Jun S, Kim KB, Park KS, Park JI. CRACD loss induces neuroendocrine cell plasticity of lung adenocarcinoma. Cell Rep. 2024;43(6):114286. Epub 20240525. doi: 10.1016/j.celrep.2024.114286. PubMed PMID: 38796854; PMCID: PMC11216895.
