# Supplementary material for: Actin dysregulation induces neuroendocrine plasticity and immune evasion: a vulnerability of small cell lung cancer
Source: Nat Commun. 2025 Dec 6;17:386. doi: 10.1038/s41467-025-67078-9 (PMC12795832; doi:10.1038/s41467-025-67078-9)
Supplement: Supplementary file 4 — Reporting Summary [file 41467_2025_67078_MOESM4_ESM.pdf]

Corresponding author(s): Kwon-Sik Park, Jae-Il Park,

Last updated by author(s): Oct 21, 2025

Reporting Summary

Nature Portfolio wishes to improve the reproducibility of the work that we publish. This form provides structure for consistency and transparency in reporting. For further information on Nature Portfolio policies, see our Editorial Policies and the Editorial Policy Checklist.

Statistics

For all statistical analyses, confirm that the following items are present in the figure legend, table legend, main text, or Methods section.

n/a	Confirmed	
☐	☒	The exact sample size (n) for each experimental group/condition, given as a discrete number and unit of measurement
☐	☒	A statement on whether measurements were taken from distinct samples or whether the same sample was measured repeatedly
☐	☒	The statistical test(s) used AND whether they are one- or two-sided<br>*Only common tests should be described solely by name; describe more complex techniques in the Methods section.*
☒	☐	A description of all covariates tested
☐	☒	A description of any assumptions or corrections, such as tests of normality and adjustment for multiple comparisons
☐	☒	A full description of the statistical parameters including central tendency (e.g. means) or other basic estimates (e.g. regression coefficient) AND variation (e.g. standard deviation) or associated estimates of uncertainty (e.g. confidence intervals)
☒	☐	For null hypothesis testing, the test statistic (e.g. F, t, r) with confidence intervals, effect sizes, degrees of freedom and P value noted<br>*Give P values as exact values whenever suitable.*
☒	☐	For Bayesian analysis, information on the choice of priors and Markov chain Monte Carlo settings
☒	☐	For hierarchical and complex designs, identification of the appropriate level for tests and full reporting of outcomes
☐	☒	Estimates of effect sizes (e.g. Cohen's d, Pearson's r), indicating how they were calculated

Our web collection on statistics for biologists contains articles on many of the points above.

Software and code

Policy information about availability of computer code

Data collection	Flow cytometry data were collected using Life Technologies Attune software. Images were captured using Axio Observer Z1M (Zeiss), Nikon confocal microscope, ECHO Revolve (ECHO), and Olympus IX73 (Olympus) microscopes. Image adjustment was performed using ZEN software (v3.0, blue edition, Zeiss). Sequencing data were generated using Illumina NovaSeq 6000 software (v1.5), and Xenium spatial transcriptomics data were obtained using the Xenium In Situ platform (10x Genomics).
Data analysis	Bulk RNA-seq data were analyzed using STAR (v2.7.10a), featureCounts (v2.0.6), and DESeq2 (v1.42.0). Single-cell RNA-seq data were processed and analyzed using Cell Ranger (v7.0.1, 10x Genomics), Seurat (v4.4.0), Scanpy (v1.9.3), scVelo (v0.3.2), and Dynamo (v1.3.0). Cell–cell communication was analyzed using CellChat (v1.6.1). Gene set enrichment analyses were performed with fgsea (v1.28.0). CUT&RUN data were processed with Bowtie2 (v2.4.2), Picard (v3.2.0), Samtools (v1.3), Bedtools (v2.31.1), and deepTools (v3.5.5). Spatial transcriptomics data were analyzed using Seurat (v4.4.0) and Xenium Explorer (v3.0.0, 10x Genomics). Additional analyses were performed with pandas (v2.0.3), infercnvpy (v0.1.0), and PHATE (v1.0.11). Statistical analyses were performed using GraphPad Prism (v9.4.0, Dotmatics). No custom code was developed beyond the tools cited above.

For manuscripts utilizing custom algorithms or software that are central to the research but not yet described in published literature, software must be made available to editors and reviewers. We strongly encourage code deposition in a community repository (e.g. GitHub). See the Nature Portfolio guidelines for submitting code & software for further information.

Data

Policy information about availability of data

All manuscripts must include a data availability statement. This statement should provide the following information, where applicable:

- Accession codes, unique identifiers, or web links for publicly available datasets
- A description of any restrictions on data availability
- For clinical datasets or third party data, please ensure that the statement adheres to our policy

Single-cell RNA-seq data have been deposited in the GEO database under accession number GSE218544, and CUT&RUN-seq data are available via GSE280263. Xenium spatial transcriptomics data generated in this study have been deposited in the GEO database under accession code GSE299069.

Research involving human participants, their data, or biological material

Policy information about studies with human participants or human data. See also policy information about sex, gender (identity/presentation), and sexual orientation and race, ethnicity and racism.

Reporting on sex and gender	Not applicable.
Reporting on race, ethnicity, or other socially relevant groupings	Not applicable.
Population characteristics	Not applicable.
Recruitment	Not applicable.
Ethics oversight	Not applicable.

Note that full information on the approval of the study protocol must also be provided in the manuscript.

Field-specific reporting

Please select the one below that is the best fit for your research. If you are not sure, read the appropriate sections before making your selection.

☒ Life sciences ☐ Behavioural & social sciences ☐ Ecological, evolutionary & environmental sciences

For a reference copy of the document with all sections, see nature.com/documents/nr-reporting-summary-flat.pdf

Life sciences study design

All studies must disclose on these points even when the disclosure is negative.

Sample size	Sample sizes for animal experiments and in vitro assays were chosen based on previously published studies of SCLC GEMMs and standard practices in the field, without statistical methods to predetermine sample size. The number of biological replicates per group is specified in the figure legends and was sufficient to ensure reproducibility.
Data exclusions	No data were excluded from the analyses. All collected data that passed quality control were included in the study.
Replication	All experiments presented in this study were performed using at least three biological replicates, unless otherwise stated. Xenium spatial transcriptomics experiments were conducted using two independent tumor samples per condition, and yielded consistent results. All presented findings were reproducible across biological replicates.
Randomization	Mice were randomly assigned to experimental groups to minimize selection bias. For in vitro assays, randomization was not relevant to the study design.
Blinding	Investigators were not blinded during data collection or analysis. Given the objective and quantitative nature of the data, blinding was not deemed necessary.

Reporting for specific materials, systems and methods

We require information from authors about some types of materials, experimental systems and methods used in many studies. Here, indicate whether each material, system or method listed is relevant to your study. If you are not sure if a list item applies to your research, read the appropriate section before selecting a response.

Materials & experimental systems

n/a	Involved in the study
☐	☒ Antibodies
☐	☒ Eukaryotic cell lines
☒	☐ Palaeontology and archaeology
☐	☒ Animals and other organisms
☒	☐ Clinical data
☒	☐ Dual use research of concern
☒	☐ Plants

Methods

n/a	Involved in the study
☒	☐ ChIP-seq
☐	☒ Flow cytometry
☒	☐ MRI-based neuroimaging

Antibodies

Antibodies used

KIAA1211/CRACD Atlas Antibodies HPA043249
ATOH1 Proteintech 21215-1-AP
MKi67 abcam ab16667
Phalloidin Cell Signaling Technology #13054
Chromogranin A abcam ab15160
ASCL1 Cell Signaling Technology #55467
ASCL1 abcam ab211327
CALCA Cell Signaling Technology #14959
Notch1 Cell Signaling Technology #3608
Cleaved Notch1 Cell Signaling Technology #4147
HES1 Cell Signaling Technology #11988
MHC Class I Cell Signaling Technology #35923
MHC Class I Cell Signaling Technology #76828
Cd8 Cell Signaling Technology #98941
Cd3 Cell Signaling Technology #99940
Cleaved caspase-3 Cell Signaling Technology #9664S
H3K27ac Cell Signaling Technology #8173
H3K27me2 Cell Signaling Technology #9728
H3K27me3 Cell Signaling Technology #9733
EZH2 Cell Signaling Technology #5246
YAP Cell Signaling Technology #14074
Pacific Blue anti-mouse CD4 Biolegend 116008
PE anti-mouse CD45 Biolegend 103106
FITC anti-mouse CD3 Biolegend 100306
APC anti-mouse CD8a Biolegend 100712
Alexa Fluor 647 goat anti-rabbit IgG Invitrogen A-21245
Alexa Fluor® 488 goat anti-rabbit IgG (H+L) Invitrogen A-11008
Goat anti-rabbit IgG HRP-linked Invitrogen A-11034
Goat anti-mouse IgG HRP-linked Fisher Scientific 31430
GAPDH Cell Signaling Technology #2119
b-actin Proteintech 66009-1-Ig
Tubulin Cell Signaling Technology #2144
The amount/dilution information for all antibodies has been included in the Supplementary table file.

Validation

All antibodies are well-recognized clones in the field and validated by the manufacturers.

Eukaryotic cell lines

Policy information about cell lines and Sex and Gender in Research

Cell line source(s)

Mouse SCLC cell lines (RPR2 and CRPR2; Mus musculus, male and female) were established from genetically engineered mouse models (GEMMs) as described in the Methods section. Human SCLC cell line (NCI-H2081; Homo sapiens, female; ATCC CRL-5920) and embryonic kidney cell line (HEK293T; Homo sapiens, female; ATCC CRL-3216) were obtained from the American Type Culture Collection (ATCC).

Authentication

Human cell lines were authenticated by short tandem repeat (STR) profiling by ATCC prior to use. Murine lines (RPR2 and CRPR2) were confirmed by genotyping and morphological verification.

Mycoplasma contamination

Cell lines have been routinely tested and confirmed negative for mycoplasma contamination

Commonly misidentified lines
(See ICLAC register)

No commonly misidentified cell lines listed in the ICLAC register were used in this study.

Animals and other research organisms

Policy information about studies involving animals; ARRIVE guidelines recommended for reporting animal research, and Sex and Gender in Research

Laboratory animals	C57BL/6 mice were purchased from the Jackson Laboratory. Both male and female mice were used at the ages of 10 week old.
Wild animals	This study did not use wild animals.
Reporting on sex	Both male and female mice were used.
Field-collected samples	This study did not involve any field-collected samples.
Ethics oversight	All mice were maintained in compliance with the guidelines of the Institutional Animal Care and Use Committee of the University of Texas MD Anderson Cancer Center and the University of Virginia School of Medicine. All animal procedures were performed based on the guidelines of the Association for the Assessment and Accreditation of Laboratory Animal Care and institutional (MD Anderson and the University of Virginia) approved protocols (protocol number 00002414-RN00 and 3967). This study was compliant with all relevant ethical regulations regarding animal research.

Note that full information on the approval of the study protocol must also be provided in the manuscript.

Plants

Seed stocks	Not applicable. This study did not involve plants or plant-derived materials.
Novel plant genotypes	Not applicable.
Authentication	Not applicable.

Flow Cytometry

Plots

Confirm that:

☒ The axis labels state the marker and fluorochrome used (e.g. CD4-FITC).

☒ The axis scales are clearly visible. Include numbers along axes only for bottom left plot of group (a 'group' is an analysis of identical markers).

☒ All plots are contour plots with outliers or pseudocolor plots.

☒ A numerical value for number of cells or percentage (with statistics) is provided.

Methodology

Sample preparation	Details are descried in the method section.
Instrument	Flow cytometry data were acquired using an Attune NxT Flow Cytometer (Thermo Fisher Scientific).
Software	Data were collected using Attune NxT software (Thermo Fisher Scientific) and analyzed using FlowJo software (version 10, BD Biosciences).
Cell population abundance	Cell populations were gated based on CD45+ expression.
Gating strategy	Sequential gating was applied using FSC/SSC to exclude debris and doublets, followed by $CD45^{+}$ selection for immune populations. $CD3^{+}$ T cells were subsequently separated into $CD4^{+}$ and $CD8^{+}$ subsets. Representative gating strategy and cut-off values are shown in Supplementary Figure 8.

☒ Tick this box to confirm that a figure exemplifying the gating strategy is provided in the Supplementary Information.
